# Genomics-driven drug discovery based on disease-susceptibility genes

**DOI:** 10.1186/s41232-021-00158-7

**Published:** 2021-03-10

**Authors:** Kyuto Sonehara, Yukinori Okada

**Affiliations:** 1 Department of Statistical Genetics, Osaka University Graduate School of Medicine, 2-2 Yamadaoka, Suita, 565-0871 Japan; 2 Laboratory of Statistical Immunology, Immunology Frontier Research Center (WPI-IFReC), Osaka University, Suita, 565-0871 Japan; 3 Integrated Frontier Research for Medical Science Division, Institute for Open and Transdisciplinary Research Initiatives, Osaka University, Suita, 565-0871 Japan

## Abstract

Genome-wide association studies have identified numerous disease-susceptibility genes. As knowledge of gene–disease associations accumulates, it is becoming increasingly important to translate this knowledge into clinical practice. This challenge involves finding effective drug targets and estimating their potential side effects, which often results in failure of promising clinical trials. Here, we review recent advances and future perspectives in genetics-led drug discovery, with a focus on drug repurposing, Mendelian randomization, and the use of multifaceted omics data.

## Background

Since the first completion of human genome sequencing in 2003 [1], many more attempts have been made to elucidate the relationships between human genotypes and phenotypes. One of the approaches that has been widely adopted for this purpose is the genome-wide association study (GWAS) [2, 3]. GWAS is an observational study that is designed to statistically assess associations between traits and tens of millions of genome-wide genetic variants from population samples. Due to the advancement of genotyping technology using single-nucleotide polymorphisms (SNP) microarray, more than 4000 GWASs have been reported globally at the time of this writing [4]. With the increase in the number of studies, the number of samples in each study has also increased, reaching hundreds of thousands of samples in recent years [5–9]. Although these GWASs have identified numerous trait-associated genomic loci, it is still challenging to translate these findings into clinical practice. In this review, we summarize recent advances in disease-susceptibility genes for drug discovery applications.

## Main text

### Significance of the genetic evidence for drug discovery

Despite the tremendous effort and substantial resources dedicated to biomedical research, only a handful of promising academic discoveries have led to new treatments [10]. Such a gap between basic research and clinical practice is a challenge for the entire field of biomedical research and is often referred to as the “valley of death.” One of the causes of this gap is the biological differences between human and other model organisms [11–13]. Validation in other organisms, such as mice, does not necessarily mean that the results will be replicated in humans. In addition, although validation using human samples is preferable, experiments using cell lines do not reflect systemic effects [14], and interventional clinical trials are at times ethically unfeasible. Investigating the impact of human genetic variation on phenotypes can provide insight into pathophysiology in the human body, which will lead to the discovery of true drug targets. Actually, it is known that drug targets with genetic evidence are more likely to be passed into the Phase III trial or market [15].

One effective approach for enhancing clinical practice is drug repurposing. Drug repurposing is a strategy for finding novel indications for existing approved drugs or drugs in clinical trials [16]. If the safety of the drug has already been confirmed in early-stage clinical trials for its original purpose, repurposing existing drugs requires less cost for testing the safety than developing and implementing novel drugs. The information on disease-susceptibility genes for drug repurposing has been successfully exploited. By utilizing databases of existing approved drug-target genes and protein–protein interactions (PPIs), Okada et al. demonstrated that GWAS-identified rheumatoid arthritis (RA)-susceptibility genes were significantly correlated with the targets of known RA drugs, such as TNF-inhibitors [17] and JAK inhibitors [18], via the PPI networks [19]. This study further revealed that CDK4 and CDK6, which are targets of approved cancer drugs, are potential therapeutic targets for RA. The efficacy of CDK4/6 inhibitors was experimentally validated in animal models of RA [20, 21]. In another example, Imamura et al. demonstrated a significant association in the connectivity of existing drug-target genes and biological type 2 diabetes (T2D) risk genes in PPI networks [22]. They identified KIF11 inhibitor (originally indicated for several types of cancers), GSK3B inhibitor (originally indicated for several types of cancers), and AP-1 inhibitor (originally indicated for RA) as potential candidates for a repurposed treatment of T2D.

The studies that have been described so far mainly focused on the association of genes and drugs for a single disease. The use of existing drug classification systems is a promising approach to systematically assess gene–drug associations for a wide variety of diseases. Malik et al. utilized the Anatomical Therapeutic Chemical Classification System (ATC) to extract multiple drug–disease associations [23]. ATC is a drug classification system in which drugs are classified according to the organ or system on which the active substances act and their therapeutic, pharmacological, and chemical properties. Malik et al. evaluated overlapping between GWAS-identified stroke-susceptibility genes and known drug targets, finding that stroke-risk genes are significantly targeted by drugs that are classified into ATC B: “Blood and blood forming organs,” specifically a subcategory, ATC B01: “Antithrombotic agents.” To perform the analysis as described above for a gene set, the freely available software GREP (Genome for REPositioning drugs) by Sakaue et al., is useful [24]. GREP quantifies the association of user-defined gene sets with the categories of existing drugs, such as the ATC or International Classification of Diseases diagnostic code. It further suggests drugs that have potential in repurposing to target the given gene set.

Given the need for novel drug development, prioritizing genes as therapeutic targets is a decisive step. Feng et al. devised a pipeline for this prioritization, named “priority index” (Pi), by integrating genome-scale data, disease ontologies, and PPIs [25]. To identify the genes responsible for the GWAS signals, they utilized not only disease-associated GWAS signals but also chromatin marks and expression quantitative trait locus (eQTL) signals. eQTLs are genomic loci that are associated with mRNA expression levels. Then, the list of candidate risk genes was extended to the genes that interact with the directly observed disease-susceptible genes via the PPI networks. Feng et al. applied a Pi pipeline to 16 immunologic traits, finding that 15 of these analytic results were significantly enriched in the targets of approved medications for the corresponding traits. These pioneering studies demonstrate that insight into disease-susceptibility genes are a powerful resource for more efficient drug discovery, and that integration of other biological data is also a key to drug discovery.

### Mendelian randomization for identifying drug targets

**Fig. 1:**
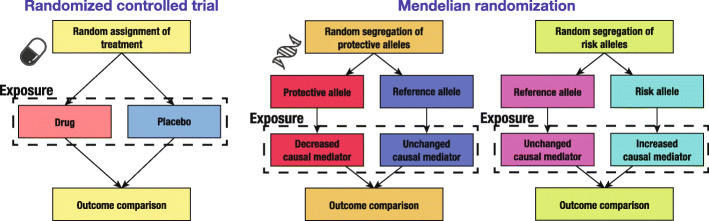
Schematic comparison between the randomized controlled trial and Mendelian randomization. Two schematic diagrams are shown for Mendelian randomization separately for the effect direction of the non-reference allele, i.e. protective or causal (risk allele). For simplicity, a single genomic locus is depicted in the diagrams of Mendelian randomization. Note that multiple loci are considered for statistical evaluation in practical settings. In RCT, random assignment of a treatment minimizes the effects of confounders. In MR, random segregation of alleles during gamete formation plays a similar role

Suitable targets for therapeutic medications are not limited to genes. Other substances, such as modified proteins or metabolites, are also related to disease states [26], and such substances are called biomarkers. However, biomarkers do not necessarily play a causal role in the disease pathology, as they can be influenced by the disease states or other causes that induce disease states. One approach to solving such a causality problem with the help of genetics is Mendelian randomization (MR) [27, 28]. MR is a genetic epidemiological framework for causal inference between an exposure (i.e., biomarker) and an outcome (i.e., disease state), as if a randomized controlled trial (RCT) had been conducted [29]. Since genotypes are assigned almost independently of environment when they are inherited from parents, those who have genotypes that increase exposure are, in effect, assigned a high dosage of the exposure, independent of other confounding factors. This situation is analogous to that of an RCT. MR provides virtual RCT opportunities without actual intervention (Fig. 1).

Sjaarda et al. conducted a systematic MR analysis of 237 biomarkers to identify the causal mediators of coronary artery disease (CAD) [30]. They found six biomarkers that are suspected to increase the risk of CAD (lipoprotein[a], apolipoprotein E, interleukin-6 receptor, stromal cell−derived factor 1 [CXCL12], apolipoprotein C3, and macrophage colony-stimulating factor 1 [CSF1]). Of these, CXCL12 and CSF1 were novel findings, and higher levels of both biomarkers were linked to an increased risk of CAD. They further utilized MR to estimate whether these candidate causal mediators affect other biomarkers of CAD risk factors, revealing an increasing effect of CSF1 on C-reactive protein levels. They inspected a causal effect of interleukin-1 beta (IL-1β) on CXCL12 and CSF1, indicating that IL-1β is causally related to CSF1 levels. As this study shows, MR analysis facilitates identification of causal relationships among various biomarkers as well as genetic variation.

Chong et al. performed a systematic MR analysis of the human proteome to identify novel causal mediators of stroke [31]. They screened 653 circulating proteins, identifying 7 potentially causal biomarkers (histo-blood group ABO system transferase, coagulation factor XI, scavenger receptor class A5 [SCARA5], tumor necrosis factor–like weak inducer of apoptosis [TNFSF12], cluster of differentiation 40, apolipoprotein[a], and matrix metalloproteinase-12). SCARA5 and TNFSF12 had an especially protective effect on cardioembolic stroke. To assess whether these two potential drug targets for stroke adversely affect other traits, Chong et al. further performed a phenome-wide MR analysis of 679 disease traits. TNFSF12 was revealed to be deleterious for four circulatory system phenotypes, three digestive phenotypes, and one injuries and poisonings phenotype, which suggests that TNFSF12-targeted treatment may cause such diseases. In contrast, SCARA5 had no significant associations with those phenotypes other than having a protective effect on subarachnoid hemorrhage. They reported SCARA5 as a promising target for the treatment of cardioembolic stroke. This study demonstrates the capability of MR to reveal novel therapeutic targets and also elucidate probable side effects.

### Future perspectives

As discussed, insight into disease-susceptibility genes are translated into clinical practice more effectively when combined with other biological resources, such as the PPI network, transcriptome, and proteome. This is because genetic information provides clues about the causal relationships among multiple traits, which can result in distinct correlations discovered from simple observational studies [32]. Hence, enhancement of multifaceted biological resources will lead to further advances in genetics-led drug discovery. For example, expansion of metabolome studies may reveal disease-causal metabolites through MR analysis, which could expand the range of candidate therapeutic targets [33]. Another potential therapeutic target is microbiota [34, 35]. The interaction between human organs and their microbial composition has received increasing attention [36]. Linking human microbial knowledge to GWAS insight is opening up new perspectives [37–39]. As previous studies show, investigating tissue-specific gene functions is an essential approach for the development of therapeutic targets [40, 41]. Integration of insight into disease-susceptibility genes and tissue-specific biological features will lead to precise strategies for treatment [42–46]. Recent advances in single-cell analysis will further reveal cell type-specific effects of genetic variation [47–49] and provide precise descriptions of relationships between genotypes and phenotypes.

Another influential factor that will provide advancements in this field is the decreasing cost of whole-genome sequencing. Nowadays, we can sequence an individual’s whole genome for less than 1000 U.S. dollars [50], which enables us to investigate the effect of rare variants on phenotypes, whereas most GWASs focus mainly on common variants. Because functional variants are subject to purifying selection, such variants tend to be rare in most populations. In other words, rare variants are more likely to be functional than common variants [51, 52]. By collecting such functional rare variants and phenotypes of carriers of those variants, we can more clearly grasp the functional effect of genotypes on phenotypes [53]. A prominent example is “human knockouts” [54, 55]. If individuals who have a homozygous loss-of-function variant within a gene are found in a population, inspecting their phenotypes closely will reveal how an individual is affected by the inhibited gene. Such observations will lead to the discovery of novel drug targets and provide an estimation of its side effects [56–58].

## Conclusions

Advances in genotyping technologies, including SNP microarray and next-generation sequencing, have yielded numerous studies concerning the relationships between the genome and a wide range of traits. The next goal for genetics is translating these insights into clinical practice. The increasing number of attempts to achieve this goal includes drug repurposing, prioritization of candidate target genes, and MR-based causal inference. Future discoveries through these efforts will lead to solutions to the present problems that challenge drug development.

## Data Availability

Not applicable.

## Abbreviations

SNP: Single-nucleotide polymorphism
PPI: Protein-protein interaction
ATC: Anatomical therapeutic chemical classification system
MR: Mendelian randomization
